# Burnout and Safety Behaviors in Maritime Operations: A Multilevel Analysis of Engagement, Quality of Life, and Work–Family Conflict

**DOI:** 10.3390/ejihpe16030039

**Published:** 2026-03-06

**Authors:** Claudio Maggio, Vittorio Edoardo Scuderi, Marcello Boccadamo, Silvia Platania

**Affiliations:** 1 Department of Educational Science, Section of Psychology, University of Catania, 95124 Catania, Italy; 2 Department of Management, Kingston University, London KT1 2EE, UK; v.scuderi@kingston.ac.uk

**Keywords:** burnout, safety behaviors, seafarers

## Abstract

Burnout represents a critical occupational health issue within the maritime sector, where demanding work schedules, prolonged periods at sea, and safety-critical responsibilities expose seafarers to significant psychological strain. This study investigates how burnout influences safety behaviors among maritime workers, adopting a multilevel framework that incorporates work engagement, quality of life, and work–family conflict as key factors shaping this relationship. Data was collected through a structured questionnaire administered to 216 seafarers distributed across 36 commercial vessels, representing a diverse range of onboard roles and operational contexts. The multilevel design allows for simultaneous examination of individual-level experiences and ship-level dynamics, offering a more nuanced understanding of how psychosocial risks translate into safety-relevant outcomes in maritime environments. Data were analyzed using multilevel structural equation modeling (MSEM), including multilevel confirmatory factor analysis (ML-CFA) and multilevel path analysis, implemented in Mplus version 8.10. The findings reveal that burnout undermines seafarers’ safe behaviors through diminished work engagement and a worsened quality of life. Furthermore, high levels of interference between work and family life amplify the negative effect of burnout on safe behaviors. This study contributes to the limited empirical literature on maritime behavioral health and provides implications for strengthening safety culture and crew well-being in the global shipping industry.

## 1. Introduction

Seafaring is widely recognized as one of the most challenging and hazardous occupational environments, where workers must cope with prolonged stays on board, separation from family and social networks, irregular shifts, and exposure to adverse physical conditions such as cold spells, hot temperatures and unstable moisture levels (Brooks & Greenberg, 2022). These stressors accumulate over time, exerting a cumulative impact on the psychological and physical well-being of maritime personnel, contributing to increased strain, emotional exhaustion and burnout (Chung et al., 2017; Lorenzi et al., 2018). Preserving seafarers’ mental health has therefore become a priority in recent years, as highlighted by the Seafarers Happiness Index report (Seafarers Happiness Index, 2025). However, despite growing awareness, recent reports indicate a decline in well-being and a heightened risk of burnout among maritime personnel. This trend is concerning since burnout could compromise their safety behaviors—both safety compliance (adherence to mandatory procedures) and safety participation (voluntary contributions to safety)—which are essential for preventing accidents at sea (Griffin & Neal, 2000; Oldenburg et al., 2013; Seafarers Happiness Index, 2025). When seafarers experience burnout, they may lack the physical and mental energy required to perform to their full potential, increasing the likelihood of errors, injuries, and fatalities (Chung et al., 2017). Between 2015 and 2024, the European Union recorded an average of 68 fatalities and 748 injuries annually, with human behavior accounting for over half of these incidents (European Maritime Safety Agency, 2025), including unsafe actions of maritime workers (Lu & Kuo, 2016). These figures underscore the urgent need to understand how burnout undermines safety in this high-risk sector.

Burnout is officially classified by the World Health Organization as an occupational phenomenon resulting from unmanaged chronic workplace stress (De Beer et al., 2024). Traditionally conceptualized by Maslach et al. (1997) along three dimensions—Emotional Exhaustion, Depersonalization, and Reduced Personal Accomplishment—the construct has been widely applied through the Maslach Burnout Inventory. In maritime contexts, several well-documented risk factors increase susceptibility to burnout: isolation and confinement, extended separation from family (Chung et al., 2017; Lorenzi et al., 2018), disruptive sleep patterns and fatigue—especially among deck and navigation officers who experience poorer sleep quality than engine personnel (Oldenburg & Jensen, 2019)—and increasing administrative and operational responsibilities, particularly for ship officers (Oldenburg et al., 2013; Baygi et al., 2022). Environmental exposures such as noise, vibration, extreme temperatures, and constant vessel movement further contribute to the erosion of psychological resources (Uğurlu et al., 2021). The absence of clear boundaries between work and private life on board amplifies these risks, as seafarers spend their limited free time in the same operational environment (Chung et al., 2017). Unlike land-based workers, traditional mechanisms for coping with burnout—such as absenteeism (Bakker & Demerouti, 2007)—are largely inaccessible at sea, where crew members cannot remove themselves from the work environment. This inability to withdraw may exacerbate chronic stress and increase vulnerability to burnout.

Conversely, work engagement represents a positive, energetic, and motivational state characterized by vigor, dedication, and absorption (Schaufeli et al., 2002; Schaufeli & Taris, 2005; Knight et al., 2017). The Broaden-and-Build Theory suggests that positive emotions associated with engagement expand cognitive repertoires and support the development of personal resources (Fredrickson, 2004), contributing to protection against burnout (Maricuțoiu et al., 2017). The Job Demands–Resources (JD-R) model similarly emphasizes how work resources such as autonomy, social support, and feedback foster engagement while mitigating burnout (Bakker et al., 2014). Engagement is strengthened by organizational factors such as supportive supervision, transformational leadership, and perceived organizational support (Knight et al., 2017), along with psychological capital resources including resilience, self-esteem, self-efficacy, and optimism (Bakker & Demerouti, 2007). Some evidence suggests that higher engagement may itself cultivate psychological capital, reinforcing a virtuous cycle of motivation and resource enhancement (De Waal & Pienaar, 2013).

In line with the Job Demands-Resources (JD-R) model, burnout among seafarers should not be considered a purely individual phenomenon, but rather the result of a complex interaction between the demands of the operating environment and the organizational resources available on board (Nagle et al., 2024). The safety climate, understood as the shared perception among crew members of how much the organization values safety, acts as a critical antecedent that shapes both compliance with procedures and active participation by workers (Gilmartin et al., 2024; Wu et al., 2008).

In this context, safety climate emerges as a critical work resource and a powerful protective factor: the shared perception that the organization values the well-being and safety of workers significantly reduces reported levels of fatigue and improves sleep quality, acting as a shield against emotional exhaustion (Hystad et al., 2017; Ma & Liao, 2025).

Alongside safety climate, supportive and safety leadership might reduce emotional exhaustion and increase psychological safety among subordinates (Dyrbye et al., 2020). Conversely, excessive workloads, chronic fatigue, and time pressures typical of navigation represent job demands that erode cognitive resources, increasing the likelihood of errors and accidents (Nagle et al., 2024). Also, characteristics of the vessel (i.e., the living and working environment of sailors) such as vibrations and noise increase physical fatigue, thus leading to poorer sleep quality and increased worker stress (Fan & Yang, 2024).

However, evidence indicates that maritime officers often display lower engagement levels than land-based professionals and have limited awareness of how to preserve their quality of life (Demir et al., 2021; Bhattacharya, 2015). According to Conservation of Resources (COR) Theory, when personal resources are depleted—such as the loss of energy caused by burnout—individuals seek to prevent further resource loss (Hobfoll, 1989; Maricuțoiu et al., 2017). Consequently, seafarers experiencing burnout may conserve their remaining energy by reducing engagement in work duties and neglecting efforts to maintain good quality of life on board. Taken together, these findings suggest that low engagement, poor quality of life, and work–family conflict may exacerbate burnout, reducing seafarers’ capacity to act safely and prevent accidents at sea. Despite these insights, little is known about how burnout interacts with engagement, quality of life, and work–family conflict to influence safety behaviors in maritime contexts. Addressing this gap is critical for developing evidence-based interventions that protect both seafarers’ well-being and operational safety.

### 1.1. Burnout and Safety Behaviors

Burnout has been identified as a key indicator of seafarers’ physical and psychological exhaustion. The absence of sufficient mental and physical strength in crew members to adhere to safety procedures and contribute to greater safety on board can result in an increased number of injuries and fatalities (Chung et al., 2017; Yuen et al., 2020). A meta-analysis by Nahrgang et al. (2011) found a positive association between burnout and the risk of accidents, injuries and fatalities in 179 articles, including adverse events such as errors and near misses. Usually, safety behaviors are positively shaped by safety climate, that is, crew members’ shared perceptions of how much the organization values and prioritizes safety (Zohar, 2003). A positive safety climate would increase their engagement and job satisfaction (Nahrgang et al., 2011) by signaling that the organization cares for employees’ well-being. This creates the potential for a virtuous cycle: workers who perceive strong managerial commitment to safety are more likely to enact safety behaviors, which in turn reinforce organizational safety practices. However, the opposite dynamic also holds as burnout undermines vigilance and cognitive control, increasing error likelihood and compromising the safety of both crew and vessel—a risk that is especially consequential in maritime operations, where human mistakes can lead to rapid and severe consequences (Lorenzi et al., 2018). Among the core dimensions of burnout, emotional exhaustion is closely tied to fatigue, defined as a state of intense tiredness or sleepiness resulting from insufficient sleep, prolonged mental or physical effort, or sustained exposure to stress (Basińska et al., 2014; Caldwell et al., 2019). Fatigue is particularly dangerous in the maritime sector, where it has been linked to reduced job security, impaired alertness, and increased risk of accidents (Wadsworth et al., 2008). On this basis, the following hypothesis is developed:

**Hypothesis** **1.** *Burnout is negatively correlated with safety behaviors*.

### 1.2. Work Engagement and Quality of Life as Mediators Between Burnout and Safety Behaviors

Although the association between seafarers’ burnout and safety behaviors is well-documented, understanding this relationship requires a theoretical framework that accounts for psychological, relational, and organizational mechanisms specific to shipboard life. Maritime work environments combine prolonged isolation, high responsibility, irregular sleep–wake cycles, and limited recovery opportunities, creating a constellation of stressors not easily captured by traditional occupational models. For this reason, the JD-R model and the COR Theory provide an essential foundation for conceptualizing the multilevel processes that link burnout to safety outcomes. The JD-R model posits two complementary pathways: a health-impairment process, where excessive job demands (e.g., workload, fatigue, emotional strain, and time pressure) erode workers’ physical and psychological resources, and a motivational process, where job resources stimulate engagement and resilience (Bakker et al., 2014). In maritime contexts, demands such as irregular shifts, sleep fragmentation caused by vessel motion, and substantial administrative and operational responsibilities—particularly among officers—create sustained strain with limited opportunities for recovery (Oldenburg et al., 2013; Oldenburg & Jensen, 2019; Baygi et al., 2022). The resulting chronic strain increases vulnerability to burnout, especially when paired with structural constraints such as limited privacy, tight living quarters, and prolonged separation from family (Chung et al., 2017; Lorenzi et al., 2018).

Psychological Capital (PsyCap), operationalized through the HERO model (Hope, Efficacy, Resilience, Optimism), acts as a key driver of safety behaviors (Caponnetto et al., 2022). Specifically, self-efficacy provides seafarers with the confidence necessary to manage risky technical tasks and strictly adhere to procedures, while resilience and optimism enable them to maintain high operational vigilance even under conditions of severe stress or chronic fatigue. Hope fuels determination to achieve safety standards, promoting not only mandatory compliance but also proactive participation in risk management on board.

HERO factors act as a buffer that protects workers from the negative effects of work demands and stress by mitigating the negative relationship between safety-related stress and safety behaviors—resilience and self-efficacy reduce the negative impact of interpersonal conflicts and excessive workload on accidents—and by making work situations seem less stressful (Margheritti et al., 2023).

Nevertheless, seafarers’ roles entail specific sources of stress linked to their job demands (Oldenburg & Jensen, 2019). For instance, officers report lower sleep quality and shorter sleep duration than engineers, often due to administrative burdens and operational responsibilities (Fan & Yang, 2024). Also, commanders and senior officers report higher levels of fatigue related to the complexity of decision-making tasks and constant pressure for operational safety (Ma & Liao, 2025; Oldenburg et al., 2013).

Integrating role stress into the resource model may clarify how psychophysical reserves are depleted differently depending on the expectations associated with the position held (Chung et al., 2017).

In parallel, the motivational process of the JD-R model highlights the role of job resources—such as autonomy, supportive supervision, constructive feedback, and developmental opportunities—in fostering work engagement. Engagement, defined as a state of vigor, dedication, and absorption (Schaufeli et al., 2002; Knight et al., 2017), supports psychological resilience and protects against the detrimental effects of prolonged demands. However, COR Theory suggests that when energy reserves are depleted by burnout, workers prioritize conserving remaining resources rather than investing in additional effort. This dynamic implies that seafarers experiencing burnout may struggle to leverage available job resources, leaving them vulnerable to further strain and less engaged (Hobfoll, 1989; Maricuțoiu et al., 2017). This is supported by empirical evidence, given that workers already experiencing burnout tend to be less energetic and engaged in their work activities over time (Hakanen & Schaufeli, 2012; Salmela-Aro & Upadyaya, 2014). More specifically, this has been observed in seafarers—particularly ship officers—as they often report lower engagement and limited awareness of health-preserving strategies, making them more susceptible to emotional exhaustion (Demir et al., 2021; Bhattacharya, 2015). Furthermore, burnout-induced resource depletion could limit workers’ capacity to maintain good standards of quality of life (Suner-Soler et al., 2013).

Within this framework, safety behaviors represent a key downstream outcome. Defined as both compliance with required safety procedures and voluntary participation in safety-improving activities (Griffin & Neal, 2000; Lorenzi et al., 2018), safety behaviors reflect the extent to which workers sustain vigilance and invest energy into minimizing operational risks. Taken together, these theoretical perspectives suggest that burnout not only undermines safety behaviors directly but also operates through low engagement and diminished quality of life dynamics in ways that may amplify risk. Yet, research has rarely examined these processes in maritime settings, where unique environmental and organizational constraints shape both demands and resources. Based on these theoretical insights and empirical evidence, the following hypotheses are presented:

**Hypothesis** **2.** *Burnout is negatively correlated with work engagement.*

**Hypothesis** **3.** *Burnout is negatively correlated with quality of life.*

**Hypothesis** **4.** *Work engagement is positively correlated with safety behaviors.*

**Hypothesis** **5.** *Quality of life is positively correlated with safety behaviors.*

**Hypothesis** **6.** *Work engagement and Quality of Life mediate the relationship between burnout and safety behaviors.*

### 1.3. Work-Family Conflict as a Moderator Between Burnout and Safety Behaviors

A further factor that may shape the negative effect of burnout on safety behavior is work-family conflict. According to Role Theory, individuals occupy multiple social roles—such as workers and parents—that shape their behaviors and expectations (Kahn et al., 1964). When these roles impose conflicting demands, individuals experience role conflict, which undermines their ability to meet expectations in either domain (Kahn et al., 1964; Nomaguchi, 2009; Reichl et al., 2014). For seafarers, prolonged absence from home, limited communication opportunities, and blurred work–life boundaries typical of shipboard life intensify emotional strain and deplete psychological resources (Chung et al., 2017; Uğurlu et al., 2021). Structural and situational factors—including long working hours, lack of private spaces for family interaction, and inflexible work shifts—further compound this challenge (Chung et al., 2017; Michel et al., 2011). These conditions make seafarers particularly vulnerable to the risk of overburdening themselves when attempting to manage competing demands from work and family life (Amstad et al., 2011; Netemeyer et al., 1996).

From the perspective of COR Theory, this dynamic reflects resource loss (Hobfoll, 1989; Maricuțoiu et al., 2017). As family strain accumulates, personal resources might be depleted, further amplifying the negative effect of burnout on safety behaviors. Over time, this erosion of resources may result in diminished vigilance, reduced compliance with safety procedures, and lower participation in safety-enhancing activities—critical behaviors in maritime operations where errors can have severe consequences (Chung et al., 2017; Lorenzi et al., 2018; Yuen et al., 2020). Given this, the following hypothesis is proposed:

**Hypothesis** **7.** *Work-family conflict moderates the relationship between burnout and safety behaviors*.

### 1.4. Gap in the Literature

While extant literature has documented the negative correlation between burnout and workplace safety, a theoretical gap remains regarding the mechanisms that underpin this relationship within the maritime environment. Specifically, these mechanisms include engagement of crew members, the quality of life in confined and high-stakes settings, and potential work-family conflicts. The present study addresses this gap by proposing a multilevel framework that moves beyond direct effects to integrate the mediating roles of work engagement and quality of life. Furthermore, by incorporating work-family conflict as a moderator, this study accounts for the complex work–life interface inherent to seafaring due to the implications of staying far from home for long periods of time.

### 1.5. Present Study and Hypotheses

Based on the theoretical foundations and empirical evidence reviewed above, it seems that burnout and safety behaviors in maritime settings emerge from a complex interplay of demands, resources, emotional experiences, and organizational conditions. Burnout is not simply the result of individual weakness, nor is safety behavior solely a matter of personal compliance. Instead, these processes unfold within a multilevel system shaped by the living-working conditions of shipboard environments, the psychological states of individual seafarers, and the broader organizational and safety climate aboard vessels. This integrated framework provides the foundation for examining how burnout, engagement, quality of life, and work–family conflict collectively influence safety behaviors in a sector where human performance is critical for preventing accidents and ensuring the well-being of crews.

This study examines how burnout influences safety behaviors among seafarers, considering both psychological mechanisms and contextual conditions that characterize maritime work. The proposed framework assumes that burnout affects safety behavior through direct and indirect pathways, and that the strength of these associations may vary depending on broader psychosocial pressures. First, burnout is expected to exert a direct negative effect on safety behaviors (H1). Higher levels of exhaustion and disengagement should reduce workers’ attention to operational details, decrease compliance with mandatory safety procedures, and weaken participation in proactive safety activities. Because burnout reflects a depletion of emotional, physical, and motivational resources, it is hypothesized to be negatively associated with work engagement (H2) and negatively associated with quality of life (H3). Seafarers experiencing strain and detachment are less likely to maintain vigor, dedication, and absorption at work, and are more prone to report dissatisfaction and diminished well-being. In contrast, both work engagement (H4) and quality of life (H5) are expected to show positive associations with safety behaviors. Engaged and satisfied individuals generally display higher motivation, attentiveness, and responsibility, making them more inclined to comply with safety standards and to contribute actively to prevention efforts.

**Figure 1:**
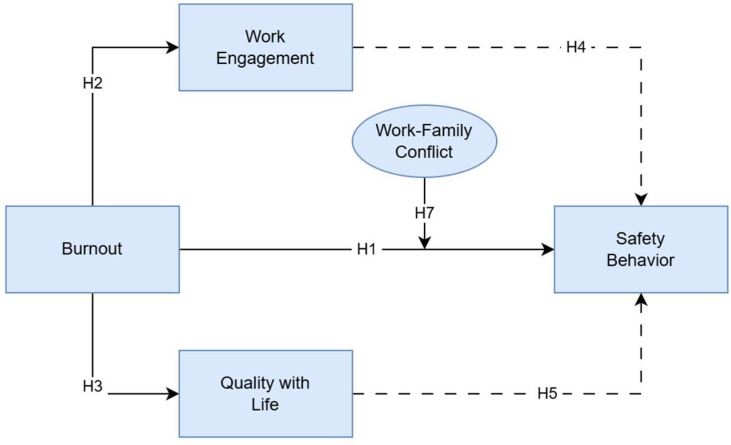
Theoretical model. Burnout is hypothesized to have a direct effect on Work Engagement (H2), on Quality with Life (H3) and on Safety Behavior (H1); Work-Family Conflict is hypothesized to moderate the effect of Burnout on Safety Behavior (H7); Work Engagement and Quality with Life are hypothesized to have a mediating effect on Safety Behavior (H4 and H5). H6 (not represented in the figure) is the combined effect of H4 and H5.

In line with these assumptions, the model proposes that the effect of burnout on safety behaviors is also indirect, unfolding through reductions in engagement and well-being (H6). When burnout erodes these psychological resources, workers’ motivational capacity and attentional focus diminish, leading to poorer safety outcomes. Finally, work–family conflict is introduced as a potential moderator (H7). Given the long periods of separation, limited contact with family, and demanding schedules typical of maritime life, work–family conflict may intensify emotional strain. It is therefore expected that the negative effect of burnout on safety behaviors will be stronger when work–family conflict is high and attenuated when conflict is low. Overall, this integrated model positions burnout as a central determinant of safety behaviors on board, while recognizing the importance of both motivational pathways (engagement), well-being resources (quality of life), and contextual pressures (work–family conflict). The conceptual structure guiding the study is illustrated in Figure 1.

## 2. Materials and Methods

### 2.1. Study Design

This study employed a cross-sectional, multilevel quantitative design to examine the relationships between burnout, work engagement, quality of life, work–family conflict, and safety behaviors among seafarers. The nested structure of the maritime work environment—where individual crew members operate within distinct vessels—made it necessary to account for potential ship-level influences when estimating the effects of psychosocial factors on safety-related outcomes.

The multilevel research design was employed to account for the nested structure of the data, where individual seafarers (Level 1) are grouped within specific vessels (Level 2); this is because seafarers working on the same ship share common environmental conditions, such as safety climates, which violates the assumption of independence required for traditional ordinary least square regression. By using a multilevel framework, we can split the variance into components that occur within and between ships, thus avoiding biased parameter estimates and preventing type 1 errors. Furthermore, from a theoretical standpoint, this design aligns with the study’s aim to capture how burnout is influenced by the shared organizational context (i.e., the vessel).

### 2.2. Participants

The sample consisted of 216 seafarers, divided among 36 ships (an average of 6 seafarers per ship). Most of the participants were male (89.9%), aged between 19 and 72, reflecting a workforce that is diverse in terms of age and experience. In terms of marital status, about half of the participants reported being married (32.4%) or cohabiting (15.7%), while the rest were single (36.6%), unmarried (9.7%), or separated/divorced (5.6%). The majority had no children, a fact that reinforces the relevance of the work-family conflict in this sector. In terms of work commitment, the duration of employment varied: 48.6% of seafarers remained on board for between 3 and 6 months, 32.4% for less than 3 months, and 19.1% for more than 6 months. More than half had international sailing experience.

The roles covered were diverse and included captains, chief engineers, deck officers, and navigation officers, allowing for analysis of the phenomenon across multiple hierarchical and functional levels. Data were collected through a structured self-report questionnaire distributed on board by trained contact officers. Before administration, captains and senior officers were informed of the study’s objectives, and authorization was obtained to conduct the survey during non-operational periods to avoid interference with safety-critical duties. Participation was entirely voluntary, and all participants provided informed consent prior to completing the questionnaire. Participants were assured of anonymity and confidentiality and completed the questionnaire individually in a private or semi-private setting. They were informed that declining or withdrawing from the study would have no consequences for their employment or professional duties. No identifiable information was collected, and responses were sealed and returned to the research team through secure channels to prevent disclosure to supervisors or ship management. The study was conducted in accordance with the Declaration of Helsinki, and the protocol was authorized by the Internal Ethics Review Board of the Department of Educational Sciences of the University of Catania (Ierb-Edunict-*2026.02.23/01*); the related research procedures followed all indications provided by the guidelines of the AIP (Italian Association of Psychology) and its Ethical Council.

### 2.3. Data Analysis

Given the hierarchical structure of the data, analyses were conducted using a multilevel structural equation modeling (MSEM) framework, which integrates multilevel confirmatory factor analysis and multilevel path modeling to simultaneously estimate measurement and structural relationships at the individual (Level 1) and vessel (Level 2) levels. This approach allowed the simultaneous estimation of individual-level relationships while accounting for ship-level variance that could influence safety behaviors or psychological states. The analysis proceeded in sequential steps, beginning with unconditional models to estimate intraclass correlations, followed by the inclusion of individual-level predictors (burnout, engagement, quality of life, work–family conflict), and finally testing cross-level effects where applicable. Statistical analyses were performed using software appropriate for multilevel modeling, and significance levels were set according to conventional standards in organizational research. The multilevel analyses were conducted using Mplus (Version 8; Muthén & Muthén, 2017).

### 2.4. Measures

To ensure methodological rigor and comparability with existing research in occupational health and safety, the study employed a set of widely validated psychometric instruments. All scales have shown satisfactory internal consistency (α > 0.70) and are commonly used in investigations involving high-risk professions, making them appropriate for application within maritime contexts. Also, all instruments used in this study showed acceptable levels of internal reliability (α > 0.70), supporting their psychometric robustness and suitability for multilevel modeling in a diverse sample of seafarers from multiple vessels.

*Burnout (BO) and Work Engagement (WE)* were assessed using the Oldenburg Burnout Inventory (OLBI) (Demerouti et al., 2001). The OLBI captures burnout through two core dimensions—Exhaustion and Disengagement. Exhaustion reflects the depletion of physical, emotional, and cognitive resources resulting from sustained work demands (e.g., “There are days when I feel tired before I arrive at work”). Disengagement represents emotional and cognitive detachment from work tasks, characterized by indifference, cynicism, or withdrawal from one’s professional role (e.g., “I am less and less interested in my work”).

A distinctive feature of the OLBI is that items are formulated in both positive and negative terms, enhancing the instrument’s ability to capture variability and reducing response biases. Moreover, this bidirectional formulation allows the OLBI to assess not only burnout but also the opposite psychological state—work engagement (Halbesleben & Demerouti, 2005; Reis et al., 2015). In this conceptualization, vigor is understood as the inverse of exhaustion, and dedication as the inverse of disengagement, aligning with the components identified by Schaufeli et al. (2002). The use of the OLBI therefore offers a more integrative assessment compared to the Maslach Burnout Inventory (MBI), addressing well-known limitations of the MBI—particularly the restricted applicability of the “personal accomplishment” dimension and its narrower focus on helping professions. Given the demanding psychosocial conditions of maritime work, where fatigue, isolation, and role overload are prominent, the OLBI provides a sound and flexible measure capable of capturing both strain and motivation within the same analytic framework.

*Quality of Life* was measured through the Satisfaction with Life Scale (SWLS) (Diener et al., 1985), a widely used instrument that assesses the cognitive component of subjective well-being. The SWLS evaluates individuals’ global judgment of their life satisfaction based on a reflective appraisal of personal circumstances relative to self-imposed standards and aspirations. This single-factor structure captures Global Life Satisfaction using items such as “If I could live my life over, I would change almost nothing.” The maritime context, characterized by long periods away from home, limited privacy, and constrained leisure opportunities, makes subjective well-being an essential variable for understanding how personal evaluations of life satisfaction may interact with burnout and engagement.

*Safety Behaviors (SB)* were measured using a questionnaire designed to assess workers’ adherence to safety procedures and their proactive engagement in safety practices. The scale captures a range of behaviors relevant to shipboard safety, including compliance with organizational protocols, consistent use of personal protective equipment, reporting of hazards or unsafe conditions, and participation in emergency drills or preparedness activities. This measure aligns with the two-component formulation of safety behavior—safety compliance and safety participation—commonly adopted in the literature on safety climate and performance (Griffin & Neal, 2000; Lorenzi et al., 2018). Considering the safety-critical nature of maritime operations, these behavioral indicators provide a meaningful proxy for understanding how psychosocial factors influence operational risk reduction.

*Work–Family Conflict (WFC)* was assessed using a scale derived from the classic framework proposed by Greenhaus and Beutell (1985). The instrument captures the extent to which demands from the work domain interfere with personal and family life. Items reflect time-based, strain-based, and behavior-based conflict, all of which are particularly relevant for seafarers who experience prolonged separation from their families, irregular work schedules, and unpredictable operational responsibilities. In maritime settings, WFC can intensify psychological strain and reduce the availability of personal resources, making it a critical construct for understanding pathways toward burnout.

## 3. Results

### 3.1. Measurement Model

Before estimating the multilevel structural relations, we evaluated the adequacy of the measurement model through a sequence of confirmatory factor analyses. A single-level CFA was first conducted to assess factorial validity at the individual level. The model demonstrated good overall fit (Table 1), with CFI and TLI values above 0.90 and an RMSEA of 0.052, indicating that the hypothesized factor structure adequately represented the data. All factor loadings were significant and ranged from moderate to high, supporting the convergent validity of the latent constructs.

To determine whether the hierarchical nature of the data required multilevel modeling, intraclass correlations (ICCs) were computed for each construct. ICC values ranged from 0.04 to 0.11, showing that a non-negligible proportion of variance was attributable to ship-level clustering. Safety Behaviors in particular displayed an ICC of 0.11, consistent with the notion that procedural adherence and safety culture may vary across vessels. These findings justified the use of a multilevel framework.

A multilevel CFA (ML-CFA) was subsequently estimated to examine the stability of the factor structure across within-ship (Level 1) and between-ship (Level 2) sources of variance. The model exhibited good fit at both levels, as shown in Table 1, with an RMSEA of 0.049 and CFI = 0.928. The SRMR within (0.045) and SRMR between (0.072) values further confirmed the adequacy of the model in reproducing covariance patterns at both levels.

Within-level loadings remained strong and consistent across constructs, indicating that burnout, engagement, quality of life, work–family conflict and safety behaviors were measured reliably at the individual level. Between-level loadings displayed greater variability, as expected given the moderate number of clusters and the inherently individual nature of certain constructs such as Quality of Life, whose between-level loadings were lower (0.33–0.64). Nonetheless, all constructs remained statistically identifiable and theoretically interpretable at both levels.

Between-level latent correlations provided additional evidence of meaningful ship-level differences. At the individual level, burnout showed strong negative correlations with both work engagement and quality of life, indicating that higher exhaustion and disengagement are systematically associated with lower motivational energy and reduced subjective well-being. In contrast, work–family conflict was positively associated with burnout and negatively associated with both engagement and safety behaviors, suggesting that interference between work and private life constitutes an additional psychosocial strain that compounds resource depletion. Engagement and quality of life were positively correlated with safety behaviors, supporting their role as protective psychological resources that foster both compliance with safety procedures and proactive participation in safety-enhancing activities. Vessels with higher average burnout tended to exhibit lower collective engagement, poorer global well-being, and weaker safety performance. Conversely, ships with more engaged crews demonstrated higher shared adherence to safety practices. These associations suggest that contextual conditions—such as organizational climate, leadership style, workload distribution, or shared safety norms—shape collective psychological states and safety outcomes.

The good fit of the measurement model at both within-ship and between-ship levels indicates that the hypothesized latent structure adequately reproduces the observed covariance patterns across the hierarchical structure of the data. This provides empirical support for the construct validity and measurement invariance of the key variables, ensuring that burnout, engagement, quality of life, work–family conflict, and safety behaviors are consistently represented at both individual and vessel levels. Consequently, the structural path estimates derived from the multilevel model can be interpreted as theoretically meaningful and not as artifacts of measurement misspecification or level-related bias.

At the individual level, burnout showed strong negative correlations with both work engagement and quality of life, indicating that higher exhaustion and disengagement are systematically associated with lower motivational energy and reduced subjective well-being. In contrast, work–family conflict was positively associated with burnout and negatively associated with both engagement and safety behaviors, suggesting that interference between work and private life constitutes an additional psychosocial strain that compounds resource depletion. Engagement and quality of life were positively correlated with safety behaviors, supporting their role as protective psychological resources that foster both compliance with safety procedures and proactive participation in safety-enhancing activities.

Taken together, the CFA and ML-CFA results confirm that the measurement model is psychometrically robust and stable across levels of analysis, providing a solid foundation for the multilevel structural equation model tested in the next stage (see Table 1).

Overall, the measurement model demonstrated strong empirical support across both hierarchical levels. The good CFA and ML-CFA fit indices, the stability of within-level loadings, the acceptable between-level measurement properties, and the meaningful between-ship latent correlations jointly indicate that the constructs were measured reliably and consistently. These results provide a methodologically robust foundation for the multilevel structural equation model reported in the following section (Table 2).

### 3.2. Structural Model

Following the establishment of a satisfactory multilevel measurement model, a multilevel structural equation model was estimated to test the hypothesized relationships among burnout, work engagement, quality of life, work–family conflict, and safety behaviors. The model exhibited good overall fit (χ^2^ = 614.52, df = 402, CFI = 0.921, TLI = 0.907, RMSEA = 0.051, SRMRwithin = 0.049, SRMRbetween = 0.067), indicating that the structural paths accurately reproduced both within- and between-level covariance patterns.

### 3.3. Direct Effects

**Figure 2:**
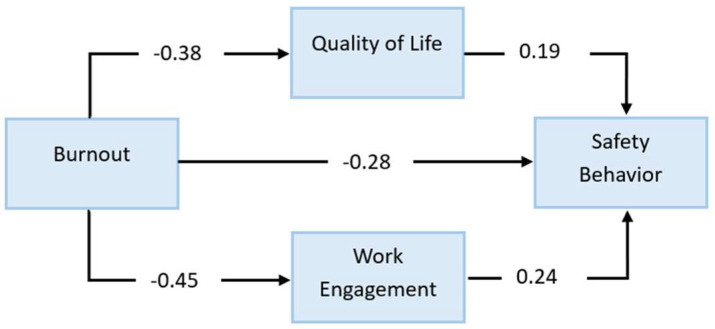
Structural Model—Direct effects.

Burnout emerged as a central determinant of safety outcomes. As shown in Table 3 and Figure 2, higher levels of burnout were associated with significantly lower levels of Safety Behaviors (β = −0.28, SE = 0.07, *p* < 0.001). This confirms that emotional exhaustion and disengagement undermine workers’ ability to comply with safety procedures and participate in safety-enhancing activities. Burnout also displayed strong negative associations with Work Engagement (β = −0.45, SE = 0.06, *p* < 0.001) and Quality of Life (β = −0.38, SE = 0.07, *p* < 0.001), supporting the view that chronic strain erodes key psychological resources. In turn, both mediating variables had positive effects on Safety Behaviors: Work Engagement (β = 0.24, SE = 0.08, *p* = 0.003) and Quality of Life (β = 0.19, SE = 0.07, *p* = 0.006) contributed uniquely to fostering compliance, attentiveness, and proactive safety involvement. These findings indicate that burnout affects safety both directly and indirectly through its impact on engagement and perceived well-being.

### 3.4. Indirect Effects

The dual mediation model was supported by significant indirect effects. The indirect effect of burnout on safety through engagement was statistically significant (indirect effect = −0.11, 95% CI [−0.18, −0.05]), suggesting that a substantial portion of burnout’s detrimental impact operates by diminishing workers’ energetic and motivational involvement in their tasks. A parallel indirect effect emerged through Quality of Life (indirect effect = −0.07, 95% CI [−0.13, −0.02]), indicating that reductions in general well-being further weaken adherence to safety practices.

These combined effects align with the Job Demands–Resources (JD-R) framework, according to which burnout depletes essential psychological resources that sustain safe and effective performance.

### 3.5. Moderation Effect of Work–Family Conflict

As hypothesized, Work–Family Conflict moderated the relationship between Burnout and Safety Behaviors (β = −0.14, SE = 0.05, *p* = 0.008). Simple slope analyses showed that when WFC was low (−1 SD), the negative association between burnout and safety was attenuated (β = −0.18). Conversely, when WFC was high (+1 SD), the effect nearly doubled (β = −0.39), indicating a marked amplification of burnout’s harmful consequences (Table 4). This pattern illustrates that seafarers who experience stronger interference between work and private life—often a chronic condition at sea—are increasingly vulnerable to the safety-impairing effects of burnout.

The structural model reveals a coherent and theoretically grounded pattern: burnout exerts a powerful influence on safety, not only through its direct effect but also through the erosion of motivational and well-being resources. Engagement and Quality of Life operate as protective factors, partially buffering burnout’s negative impact, while Work–Family Conflict exacerbates vulnerabilities, making safety performance particularly fragile in situations of strong home–work interference. These findings underscore the importance of interventions that address both organizational conditions contributing to burnout and psychosocial resources that sustain safe performance on board. From a process perspective, this pattern suggests that work–family conflict intensifies the safety-impairing effects of burnout by further depleting attentional and self-regulatory resources. When cognitive and emotional resources are taxed by competing role demands, seafarers are more likely to experience reduced situational awareness, impaired risk perception, and lower capacity to sustain proactive safety behaviors in high-demand operational settings.

## 4. Discussion

The present study examined the multilevel relationships between burnout, work engagement, quality of life, work–family conflict, and safety behaviors among seafarers. The findings consistently indicate that burnout is a central risk factor for safety performance on board, exerting both direct and indirect detrimental effects. Seafarers experiencing higher levels of exhaustion and disengagement were significantly less likely to comply with safety procedures or participate in proactive safety practices (H1), confirming prior research illustrating how stress, fatigue, and emotional depletion undermine situational awareness and operational readiness (Oldenburg & Jensen, 2019; Oldenburg et al., 2009; Uğurlu et al., 2021).

Beyond documenting significant associations, the present findings help clarify the psychological and organizational mechanisms through which burnout translates into diminished safety performance in maritime settings. At the individual level, resource depletion processes—manifested in reduced motivational energy, attentional capacity, and self-regulatory control—undermine seafarers’ ability to maintain vigilance and proactive engagement in safety-related tasks. At the collective level, ship-specific contextual conditions, such as leadership practices, shared safety norms, and psychosocial climate, appear to shape how these individual resource losses accumulate into vessel-level patterns of engagement and safety performance.

Burnout also showed strong negative associations with work engagement (H2) and quality of life (H3), echoing prior meta-analytic evidence that engagement and burnout operate as distinct yet interrelated psychological states, often reinforcing each other over time (Maricuțoiu et al., 2017; Platania et al., 2022a, 2022b). In the maritime context, where work schedules are irregular, sleep quality is frequently impaired, and exposure to psychosocial stressors is common (Baygi et al., 2022), these dynamics appear particularly pronounced. The decline in engagement is concerning, given that engagement has been linked to enhanced resilience, proactive safety involvement, and improved performance across high-demand sectors (Knight et al., 2017; Platania et al., 2022b; Caponnetto et al., 2022). Also, work engagement (H4) and quality of life (H5) reported positive and significant effects on safety behaviors.

The mediating roles of engagement and quality of life underscore the broader mechanism through which burnout compromises safety performance (H6). Rather than exerting its influence only via emotional fatigue, burnout progressively erodes motivational energy and psychological well-being, weakening workers’ capacity to remain attentive and compliant during safety-critical operations. The dual mediation pattern aligns with JD-R theory and COR theory, demonstrating how psychological resources operate as buffers against operational risk.

Work–family conflict further amplified this vulnerability (H7). The negative association between burnout and safety behaviors was significantly stronger among seafarers reporting high WFC. This finding resonates with existing evidence showing that extended time at sea, prolonged separations from family, and limited contact with social support systems create structural barriers to work–life balance in maritime work (Baygi et al., 2022; Oldenburg & Jensen, 2019). When personal and occupational strain converge, seafarers may have fewer psychological resources available to sustain safe behaviors.

Finally, the multilevel results showed meaningful between-ship differences in shared levels of burnout, engagement, and safety behaviors. This is consistent with research demonstrating that safety climate, leadership style, and psychosocial safety climate can differ across organizational units and influence collective performance (Platania et al., 2022b). Although individual factors remain central, these findings suggest that some vessels may operate under more protective psychosocial conditions than others.

Overall, the results reinforce that burnout is not merely an individual psychological experience, but a systemic risk factor embedded within the operational, organizational, and psychosocial realities of maritime work. Models of maritime safety should therefore incorporate psychological strain variables alongside more traditional technical and procedural indicators.

### 4.1. Theoretical Implications

This study makes a theoretical contribution by exploring the mechanisms through which burnout could compromise safety behaviors of seafarers in the maritime context. Firstly, applying the JD-R model and COR theory to this context confirms that resource depletion and motivational decline are not isolated processes but operate in tandem to influence safety behaviors. The dual mediation effect of work engagement and quality of life demonstrates that burnout compromises safety performance through both motivational and well-being pathways. This reinforces the dual-process perspective of the JD-R model and extends the resource loss spiral of COR theory to environments characterized by prolonged isolation and limited recovery opportunities. Secondly, the moderating role of work–family conflict highlights the need to integrate role-based stressors into resource models. This finding suggests that personal and occupational demands interact to amplify resource depletion. So, existing frameworks should consider cross-domain stressors—such as work-family conflicts—when predicting safety outcomes in high-risk settings. Thirdly, the multilevel results emphasize that burnout is not merely an individual phenomenon but is also influenced by organizational and psychosocial factors. Differences between ships in shared levels of burnout, engagement and safety behaviors highlight the importance of collective resources and safety climate. This suggests that JD-R and COR models should incorporate group-level dynamics to fully capture safety risk processes. Lastly, by linking burnout and safety behavior through work engagement and quality of life, this study demonstrates that these psychological resources are not only indicators of well-being but also determinants of compliance and proactive safety behaviors. This theoretical integration calls for a broader conceptualization of safety models, incorporating psychological strain variables.

From a cumulative and interactive perspective, burnout among seafarers emerges not as the result of a single dominant stressor, but as the outcome of a dynamic interplay between sustained job demands and progressively depleted resources. High operational demands—such as irregular shifts, sleep disruption, prolonged confinement, and role overload—interact with limited recovery opportunities, reduced social support, and persistent work–family conflict. As engagement and quality of life decline, seafarers enter a resource loss spiral, as described by Conservation of Resources theory, in which diminished psychological and emotional resources reduce their capacity to cope with ongoing demands, thereby further intensifying burnout. This cumulative process underscores the need to conceptualize burnout as a systemic and interactive phenomenon rather than a purely individual response to isolated stressors

### 4.2. Practical Implications

The findings of this study offer several important implications for maritime organizations and shipping companies committed to improving safety performance and safeguarding crew well-being. The evidence suggests that burnout must be treated not merely as an individual health concern but as a core operational risk that directly undermines safety behaviors on board. Given that exhaustion and disengagement compromise vigilance, situational awareness, and compliance with procedures, companies should incorporate psychological strain indicators into routine safety monitoring. Regular assessments using validated instruments could help identify vessels or workgroups where psychosocial conditions are deteriorating and where safety performance may consequently be at risk.

The strong protective roles of work engagement and quality of life further indicate that addressing crew well-being has tangible operational benefits. Enhancing engagement—through supportive leadership, constructive feedback, opportunities for participation, and clear communication—can strengthen the psychological resources that sustain safe behaviors even under demanding conditions. Likewise, interventions aimed at improving daily living conditions on board, such as access to recreational activities, better sleep environments, healthier work–rest cycles, and more stable opportunities for communication with families, may contribute substantially to safer performance.

The moderating effect of work–family conflict highlights a structural challenge in maritime work: prolonged separation from family and limited opportunities for personal recovery intensify the consequences of burnout. This finding suggests that organizational policies related to crew rotation, contract duration, shore leave predictability, and communication technologies should be carefully evaluated. Reducing the chronic strain associated with work–family interference may help buffer the negative effects of burnout on safety and improve overall well-being.

Also, the multilevel results demonstrate that ships differ meaningfully in shared psychosocial conditions, indicating that vessel-level culture and leadership practices play a pivotal role. Shipping companies may therefore benefit from strengthening the psychosocial safety climate at the ship level. For example, businesses could train captains and officers in supportive leadership, fostering an open safety dialogue, and ensuring that organizational values related to safety and crew care are consistently communicated and enacted across vessels. By integrating psychosocial considerations within safety management systems, organizations can promote a more resilient and safety-oriented working environment.

In terms of potential intervention measures in the maritime context, it is recommended to adopt a multi-level strategy integrating organizational changes, leadership support, and individual resources. At the organizational level, companies should adopt regular psychosocial health monitoring systems using validated tools to identify early on the units (ships) most at risk of burnout. Structural interventions should aim to optimize work-rest cycles and improve living conditions on board, for example by ensuring stable access to communication technologies to mitigate isolation and work-family conflict. With regard to leadership, it is crucial to train commanders and officers in a supportive style that fosters a climate of psychological safety, where seafarers feel free to report hazards without fear of punishment. This approach includes providing constructive feedback and formally recognizing proactive safety behaviors, thereby strengthening the bond of trust between the crew and management. At the individual level, safety training programs should be supplemented with resilience-building and mental health support interventions aimed at strengthening workers’ psychological capital. Furthermore, designing customized interventions based on role-specific burnout profiles can ensure a more efficient distribution of support resources, while simultaneously improving staff well-being and the reliability of maritime operations.

### 4.3. Limitations and Directions for Future Research

Several limitations should be acknowledged when interpreting the findings of this study. Firstly, cross-sectional design limits the ability to draw firm causal conclusions. Although the structural model is theoretically grounded and supported by empirical evidence, longitudinal research is needed to determine whether burnout precedes changes in engagement, well-being, and safety behaviors over time. Secondly, all variables were assessed through self-report measures, raising the possibility of common method bias and social desirability effects. While the use of validated instruments and multilevel modeling helps mitigate this concern, future studies should incorporate objective safety indicators, behavioral observations, or supervisor ratings to triangulate the results. Thirdly, although the sample included 216 seafarers across 36 ships, the number of clusters remains modest for detecting more complex between-level dynamics. Some constructs—such as quality of life—displayed lower between-ship reliability, suggesting that larger samples or designs explicitly optimized for detecting vessel-level variability would strengthen future multilevel analyses. Fourthly, the study did not examine specific organizational or environmental factors that might contribute to ship-level differences in burnout and safety behaviors, such as leadership style, safety climate, vessel type, voyage patterns, or workload conditions. Incorporating these variables would provide a clearer understanding of how operational contexts shape psychosocial experiences and safety outcomes. Lastly, the study focused exclusively on merchant seafarers; caution is needed in generalizing the results to other maritime sectors such as offshore, fishing, cruise, and military operations, where working conditions and psychosocial dynamics may differ substantially. Despite these limitations, the study contributes meaningful evidence to the maritime safety literature and highlights important avenues for future research.

## 5. Conclusions

This study shows that burnout is a significant determinant of safety behaviors among seafarers, influencing performance both directly and indirectly through its effects on engagement and quality of life. Work–family conflict further exacerbates this vulnerability, particularly in a sector characterized by isolation, demanding schedules, and prolonged periods away from home (Oldenburg & Jensen, 2019; Baygi et al., 2022). The multilevel findings reveal that safety performance is shaped not only by individual strain but also by differences in ship-level psychosocial climate and working conditions. Taken together, these results highlight the need for shipping companies to adopt an integrated approach to safety management that includes psychological well-being as a core component. Enhancing engagement, improving life conditions on board, and reducing work–family interference may substantially mitigate burnout’s negative effects and foster safer operational practices at sea.

The multilevel findings indicate that neither individual-level strain nor ship-level psychosocial conditions alone can fully account for safety performance. Individual-level burnout, engagement, and quality of life explain a substantial proportion of variance in safety behaviors, highlighting the central role of personal psychological resources. However, the presence of meaningful between-ship variance and significant ship-level latent correlations suggests that shared contextual factors—such as leadership style, safety climate, and organizational norms—systematically shape collective levels of strain, motivation, and safety practices. Rather than one level being inherently more prominent, the results support an interactive perspective in which individual psychological processes unfold within, and are either amplified or buffered by, vessel-level organizational and psychosocial conditions.

Future research should examine these relationships longitudinally, evaluate targeted interventions to reduce burnout and strengthen engagement, and explore how ship-level organizational factors—such as leadership, crew cohesion, and psychosocial safety climate—shape collective safety outcomes and accident prevention at sea. Investing in the well-being of seafarers is equivalent to investing in the safety of the entire maritime system: safer ships, healthier crews, and more resilient organizations.

## Figures and Tables

**Table 1:** CFA and Multilevel CFA Fit Indices and Intraclass Correlations.

Model/Statistic	Value
***Single-level CFA Fit***	
*χ*^2^(312)	486.41
*CFI*	0.936
*TLI*	0.924
*RMSEA* (90% CI)	0.052 [0.044, 0.059]
*SRMR*	0.047
***Intraclass Correlations (ICCs)***	
*Burnout*	0.07
*Work Engagement*	0.05
*Quality of Life*	0.04
*Work–Family Conflict*	0.06
*Safety Behaviors*	0.11
***Multilevel CFA Fit***	
*χ*^2^(356)	512.73
*CFI*	0.928
*TLI*	0.915
*RMSEA* (90% CI)	0.049 [0.041, 0.056]
*SRMRwithin*	0.045
*SRMRbetween*	0.072

**Table 2:** Standardized Factor Loadings (Within and Between Levels) and Between-Level Latent Correlations.

Construct	Within Loadings	Between Loadings
*Burnout*	0.58–0.81	0.49–0.77
*Work Engagement*	0.74–0.86	0.52–0.75
*Quality of Life*	0.62–0.80	0.33–0.64
*Work–Family Conflict*	0.55–0.78	0.45–0.71
*Safety Behaviors*	0.68–0.84	0.57–0.81
***Between-Level Latent Correlations***		
*Burnout ↔ Safety Behaviors*		−0.41
*Burnout ↔ Engagement*		−0.52
*Burnout ↔ Quality of Life*		−0.47
*Engagement ↔ Safety Behaviors*		0.36
*Quality of Life ↔ Safety Behaviors*		0.29

**Table 3:** Direct and Moderated Effects in the Multilevel Structural Model.

Path	β	SE	*p*
*Burnout → Safety Behaviors*	−0.28	0.07	<0.001
*Burnout → Work Engagement*	−0.45	0.06	<0.001
*Burnout → Quality of Life*	−0.38	0.07	<0.001
*Work Engagement → Safety Behaviors*	0.24	0.08	0.003
*Quality of Life → Safety Behaviors*	0.19	0.07	0.006
*Burnout × WFC → Safety Behaviors*	−0.14	0.05	0.008

**Table 4:** Indirect and Moderated Effect.

Effect	Estimate	95% CI
*Burnout → Work Engagement → Safety Behaviors*	−0.11	[−0.18, −0.05]
*Burnout → Quality of Life → Safety Behaviors*	−0.07	[−0.13, −0.02]
*Simple slope (Low WFC, −1 SD)*	−0.18	—
*Simple slope (High WFC, +1 SD)*	−0.39	—

## Data Availability

The original contributions presented in this study are included in the article. Further inquiries can be directed to the corresponding author.

## Abbreviations

The following abbreviations are used in this manuscript:

JDR	Job Demands–Resources
COR	Conservation of Resources
OLBI	Oldenburg Burnout Inventory
BO	Burnout
WE	Work Engagement
SWLS	Satisfaction with Life Scale
SB	Safety Behaviors
WFC	Work-Family Conflict
ICCs	Intraclass Correlations
CFA	Confirmatory Factor Analysis
